# Characterisation of integrin-linked kinase signalling in sporadic human colon cancer

**DOI:** 10.1038/sj.bjc.6600939

**Published:** 2003-05-27

**Authors:** A Marotta, K Parhar, D Owen, S Dedhar, B Salh

**Affiliations:** 1 Jack Bell Research Center, 2660 Oak Street, Vancouver, BC, Canada V6H 3Z6; 2 Vancouver General Hospital, 855 W12th Ave, Vancouver, BC, Canada V5Z 1M9; 3 BC Cancer Agency, 600 W10th Ave, Vancouver, BC, Canada V5Z 4E6

**Keywords:** colon cancer, ILK, *β*-catenin

## Abstract

The putative oncogene, integrin-linked kinase (ILK) is a protein serine/threonine kinase that has been reported to regulate a number of biological properties including anchorage-independent cell cycle progression, tumour cell invasion and apoptosis. Overexpression of ILK has been documented in a wide variety of human malignancies including Ewing's sarcoma (ES), primitive neural ectodermal tumours (PNETs) and prostate tumours (PT). We recently reported that ILK signalling was also dysregulated in patients with the genetic condition familial adenomatous polyposis (FAP), a precursor to colon cancer. In this study, we extended our previous work by investigating the ILK-signalling pathway in sporadic human colon cancer and representative lymph node metastases. The data indicate that the ILK protein is significantly hyperexpressed in malignant acini in relation to normal crypts. Moreover, overexpression of ILK not only coincided with increased MBP phosphotransferase activity but as well with effects on downstream targets like GSK3*β*. Based upon the presented data, we propose that ILK signalling is dysregulated early during the development of human colon cancer, and that selective inhibition of this molecule alone or in combination with the standard therapeutic modality might be a more effective means of treating colon cancer.

Mutation of the adenomatous polyposis coli (APC) gene is an integral event in the genesis of colorectal cancer (Fearon *et al*, 1990; Kinzler *et al*, 1991). Mutation of this gene results in the expression of a C-terminally truncated protein that is unable to form a complex with axin, *β*-catenin and GSK3*β* (Behrens *et al*, 1998; Sparks *et al*, 1998; Barker *et al*, 2000; Rowan *et al*, 2000). Consequently, there is an increase in the cytosolic levels of *β*-catenin. Stabilisation of the latter is believed to result in its translocation to the nucleus where it binds to the Tcf-4 (T-cell factor) family of transcription factors resulting in the expression of a number of different genes that have been implicated in oncogenesis. These include cyclin D1, c-myc and the matrix metalloproteinase (MMP)-7 (He *et al*, 1998; Crawford *et al*, 1999; Shtutman *et al*, 1999). However, whether mutation of APC alone is sufficient in dysregulating *β*-catenin signalling or whether additional signals are required for this disruption are currently unclear. In this regard, a prominent nuclear *β*-catenin signal was documented in cells that overexpress the integrin-linked kinase (ILK), duplicating the events associated with the mutation of APC. Translocation of *β*-catenin accompanied the activation of Tcf-4-dependent gene transcription (Novak *et al*, 1998).

The ILK, which was discovered through its interactions with the *β*1 integrin subunit (Hannigan *et al*, 1996), has been demonstrated to mediate a plethora of biological events. This putative oncogene has not only been described as an immunohistochemical marker for the identification of Ewing's sarcoma (ES) and primitive neuroectodermal tumours (PNET), but as well increased expression of the protein has been demonstrated to be inversely related to the 5-year survival rate in prostate cancer (Chung *et al*, 1998; Graff *et al*, 2001). In addition to this, we demonstrated that ILK signalling is dysregulated in patients diagnosed with familial adenomatous polyposis (FAP) (Marotta *et al*, 2001). In the present study, we sought to determine the extent to which this pathway was disrupted in sporadic cases of colon cancer. The results from these studies demonstrate that ILK is hyperexpressed in malignant crypts from both the primary and metastatic lesions. In addition to this, we demonstrate that there was approximately a 2–9-fold increase in ILK immunoprecipitated MBP phosphotransferase activity in relation to normal colonic crypts. Furthermore, we report that changes in ILK activity coincide with changes on downstream targets, primarily GSK3*β*. Based upon our findings, we conclude that dysregulation of the ILK-signalling nexus is an important early event in the genesis of human colon cancer.

## MATERIALS AND METHODS

### Materials

Rabbit polyclonal antibodies for ILK (IB/IHC), PKB and GSK3*β* were kindly provided by Stressgen Biotechnologies Inc. (Victoria, BC, Canada). Monoclonal anti-ILK (IP) and MBP were obtained from Upstate Biotechnology Inc. (Lake Placid, NY, USA). Anti-phosphospecific-PKB Ser473 and GSK3*β* Ser-9 were obtained from New England Biolabs Inc. (Beverly, MA, USA). Horse-radish peroxidase-conjugated secondary antibodies were obtained through Calbiochem (San Diego, CA, USA). cAMP-dependent protein kinase inhibitor peptide, EGTA, EDTA, MOPS, PMSF, sodium orthovanadate, leupeptin, aprotinin, benzamidine, dithiothreitol and *β*-glycerolphosphate were purchased from Sigma, Sigma-Aldrich, Oakville, Ontario.

### Tissue procurement

We obtained a total of 38 cases of human colon cancer through Dr D Owen from the Division of Anatomical Pathology at Vancouver Hospital and Health Sciences Centre (VH&HSC). The 38-paired cases were used for biochemical analysis. In all, 16 of the cases, which were utilised in these studies, were selected for immunohistochemical analysis on the basis that lymph node metastases were present. Ethical consent for these studies was obtained from each of the patients and by boards governing research at the University of British Columbia and VH&HSC.

### Preparation of human tissue samples

The tissue samples were serially sectioned (3–5 *μ*m in diameter) using a cryostat, and approximately 20 slices were placed in 1 ml of homogenisation buffer containing 20 mM MOPS, 50 mM *β*-glycerolphosphate, 50 mM sodium fluoride, 1 mM sodium vanadate, 5 mM EGTA, 2 mM EDTA, 1% NP40, 1 mM dithiothreitol, 1 mM benzamidine, 1 mM phenylmethanesulphonylfluoride and 10 *μ*g ml^−1^ leupeptin as previously described (Marotta *et al*, 2001).

### Sodium dodecylsulphate–polyacrylamide gel electrophoresis (SDS–PAGE)

Protein samples for immunoblotting were resolved using SDS–PAGE. Proteins were transferred onto the nitrocellulose membrane in a BioRad transfer apparatus and the nitrocellulose membrane was blotted with the appropriate antibody at a dilution of 1 : 1000 in 0.05% Tween-TBS. The resulting membrane was exposed to ECL for 1 min, and then to film to visualize the immunoreactive proteins.

### Immunoprecipitation

A total of 400 *μ*g of the appropriate sample was subjected to a preclear step with a nonspecific rabbit Ig G antibody preabsorbed to protein A Sepharose for a minimum of 1 h at 4°C. The samples were then centrifuged at 6000 r.p.m. and equal volumes of the supernatent were taken and aliquoted into a new microfuge tube. The lysate was then incubated with 4 *μ*g of the appropriate antibody overnight at 4°C with gentle mixing. To each vial, 30 *μ*l protein A Sepharose was added for an additional 1 h at 4°C. The lysates were then centrifuged and the supernatent was discarded. The protein A Sepharose beads conjugated to the antibody were washed twice with the standard lysis buffer and twice with protein kinase reaction buffer (100 mM Hepes pH 7.0, 2 mM MgCl_2_, 2 mM MnCl_2_ and 2 mM Na orthovanadate).

### Immune complex kinase assays

The beads were pelleted and the reaction was started by the addition of 25 *μ*l of the kinase reaction buffer (50 mM Hepes pH 7.0, 1 mM MgCl_2_, 1 mM MnCl_2_, and 1 mM Na orthovanadate), 2 mM NaF, 5 *μ*g of MBP per tube and 0.5 *μ*g of ATP (250 *μ*M ATP, 1 *μ*Ci[*γ*-^32^P]ATP) at 30°C for 20 min. The reaction was terminated with the addition of 10 *μ*l of 4 × sample buffer. The tubes were microfuged at maximum for 1 min and the proteins were resolved on a 14% SDS–PAGE gel. The resulting gel was stained with Coomassie Blue as outlined above and phosphorylation of the substrate was visualised by autoradiography.

### Immunohistochemistry

Formalin-fixed paraffin-embedded tissue sections were obtained through the Division of Anatomical Pathology at VH&HSC. Immunohistochemical staining for ILK (1 : 100) and phospho-Ser473 PKB (1 : 100) was carried out using a standard strepta-vidin–biotin technique. Antigen retrieval was performed by pressure-cooking the sections in a 0.01 M citrate buffer for 10 min at high power using a 925-W microwave oven. The slides were then incubated with the appropriate primary antibody at a pre-determined concentration overnight at room temperature. Following incubation, the sections were rinsed three consecutive times with PBS and then incubated with the appropriate biotinylated secondary antibody for 1 h followed by incubation with peroxidase-labelled streptavidin. AEC substrate was used as the chromagen and the sections were counterstained with haematoxylin. Three observers independently examined all the stained immunoreactive positivity was assessed by uniform red staining.

The staining intensity (weak=1, intermediate=2 and strong=3) was scored by three independent examiners (AM, DO and BS), and the results are expressed as the mean±s.d.

### Statistical analysis

The relative amounts of protein were measured by scanning the film using the BioRad gel-doc apparatus into a TIFF format file. The numerical densitometric values were assigned arbitary values on a scale of 1–3 for immunohistochemistry. The band densities and the results are expressed as mean±s.d., with *P*<0.05 being considered significant using the Student's *t*-test (unpaired, two-tailed).

## RESULTS

### ILK expression is dysregulated in sporadic cases of colon cancer

A recent report from our laboratory indicated that the expression and activity of ILK is perturbed in polypoid lesions resected from patients diagnosed with the autosomal dominant condition, FAP (Marotta *et al*, 2001). To determine whether ILK was also dysregulated in sporadic cases of human colon cancer, we examined both the protein expression of ILK by immunohistochemical analysis and the mRNA levels using microarray technology.

**Figure 1:**
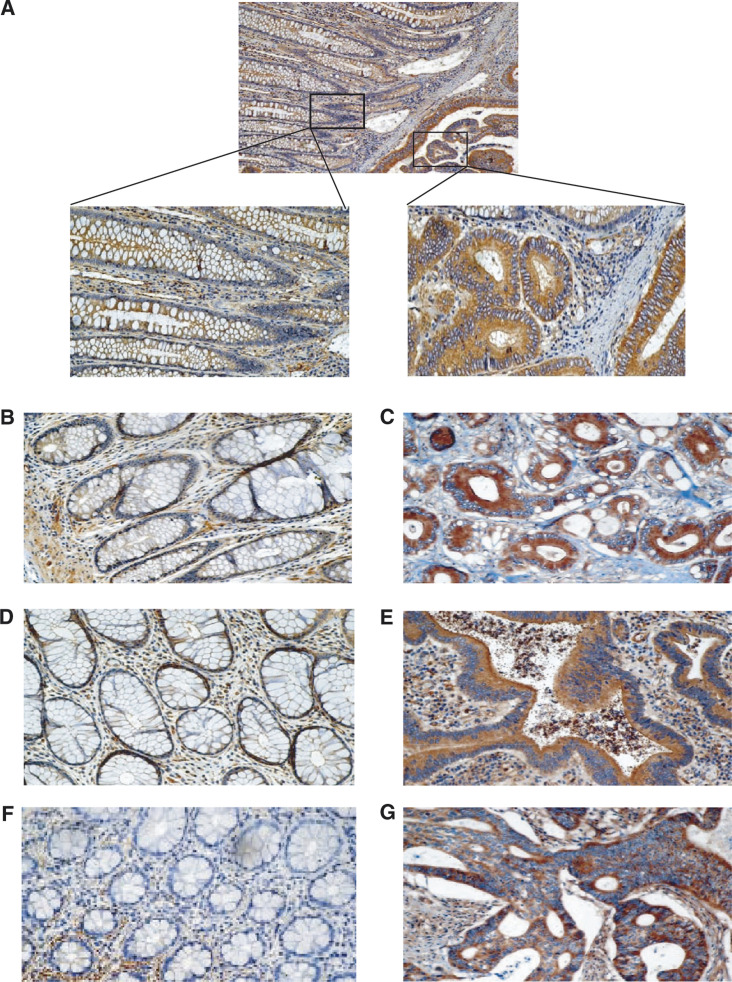
Over-expression of ILK in sporadic colorectal cancers. Panel **A**: representative case examining ILK expression in the control crypts *vs* cancerous crypts at a lower magnification (× 100) as well as at a higher magnification (× 200). Panels **B**–**G**: three additional representative cases demonstrating enhanced ILK expression in the cancerous lesions (**C**, **E**, **G**) when compared with the normal control (**B**, **D**, **F**). Staining was performed as outlined in the Materials and Methods section.

Immunohistochemical analysis revealed that the protein levels of ILK were dramatically increased in the cancerous acini when compared to the normal adjacent control crypts based on the intensity of the chromagen (Figure 1A

; upper panel). This increase in the protein expression of ILK was further apparent at higher magnification (Figure 1A, lower panels). The results are corroborated by the micrographs presented in Figure 1B–G, which represent three separate cases. These results clearly demonstrate that the protein expression of ILK is increased in the cancerous crypts (panels C, E, G) with respect to the adjacent control tissue for each case (panels B, D, F).

**Figure 3:**
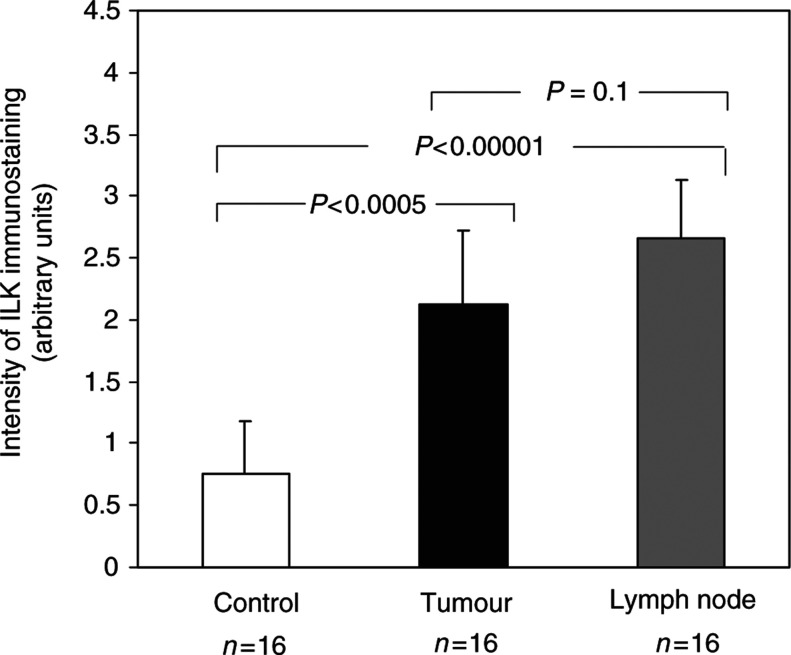
ILK expression is significantly increased in colorectal cancers. Mean expression of ILK in 16 cases of colon cancer with positive lymph nodes. The intensity of the chromagen (weak=1, moderate=2, strong=3) was scored as outlined in the Materials and Methods section by three independent examiners. The results for the 16 cases are expressed as the mean±s.d.

To determine whether overexpression of ILK within these lesions was statistically significant, the relative staining intensity for each case was scored. The data thus obtained revealed that the increase in ILK in the cancerous crypts was highly significant (*P<*0.0005). There was approximately a three-fold increase in the expression of ILK in the cancerous acini (2.22) when compared to the normal adjacent tissue (0.75, Figure 3

). It is worth noting that although ILK immunoreactivity was observed in the stromal component of both the normal and cancerous lesions, this staining was not taken into account when quantifying the intensity of the chromagen for statistical analysis. ILK immunoreactivity within the stromal component of tissue is not an unexpected finding, since this protein is ubiquitously expressed (Li *et al*, 1999). Analysis of the mRNA levels indicated that there was no significant differences in the ILK mRNA levels between any of the controls and primary lesions analysed (data not shown). Thus, it appears that the increased ILK protein expression likely reflects a change in protein stabilisation as opposed to a change in the level of the message.

### ILK expression in regional lymph nodes

A number of studies have underscored the importance of ILK in mediating cell migration and invasion. In this regard, overexpression of ILK has been reported to upregulate the levels of MMP-9 in an AP-1-dependent manner. Moreover, treatment with the selective ILK inhibitor (termed KP-SD1) was shown to inhibit MMP-9 promoter activity as well as lead to a reduction in the invasive potential of intestinal and mammary epithelial cells (Troussard *et al*, 2000).

**Figure 2:**
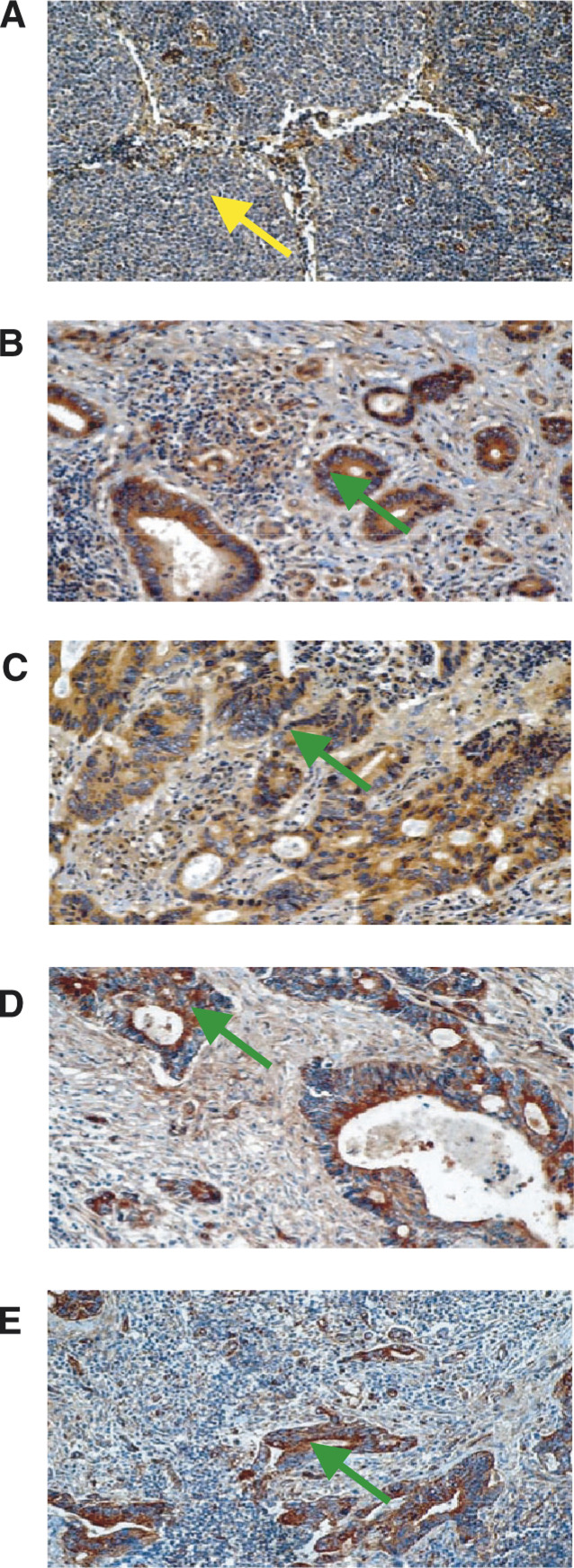
ILK expression in positive lymph nodes. Panel **A**, expression of ILK in a lymph node negative for tumour cell infiltration. Panels **B**–**E**, expression of ILK in four tumour cell positive lymph nodes. Immunohistochemical analysis of ILK was performed as outlined in the Materials and Methods section.

To characterise the protein expression of ILK in metastatic lesions, 16 cases were selected on the basis that metastatic deposits were present in the regional lymph nodes. The representative results from these studies are presented in Figure 2

. In Figure 2A, which represents a control lymph node, a moderate chromagenic signal is present in a small proportion of the lymphocytes as well as the lymphatic vessels (yellow arrow head). In Figure 2B–E, which represents four individual cases with positive lymph nodes, a strong chromagenic signal is observed predominantly in the malignant acini (green arrow heads). To delineate the extent to which the protein expression of ILK was altered during the metastatic process, the relative staining intensity was quantified and compared to the control tissue as well as the primary cancer. The results presented in Figure 3 indicate that the protein expression of ILK is significantly increased approximately four-fold in the malignant acini of the regional lymph nodes when compared to the normal colonic crypts (*P*<0.00001). There was no significant difference in the expression of ILK between the primary cancer and the metastatic deposit within regional lymph nodes (*P*=0.1). Based upon these results, it appears that changes in the expression of ILK occur prior to changes in the metastatic potential of colon cancer cells.

### ILK activity is increased in colonic cancers

**Figure 4:**
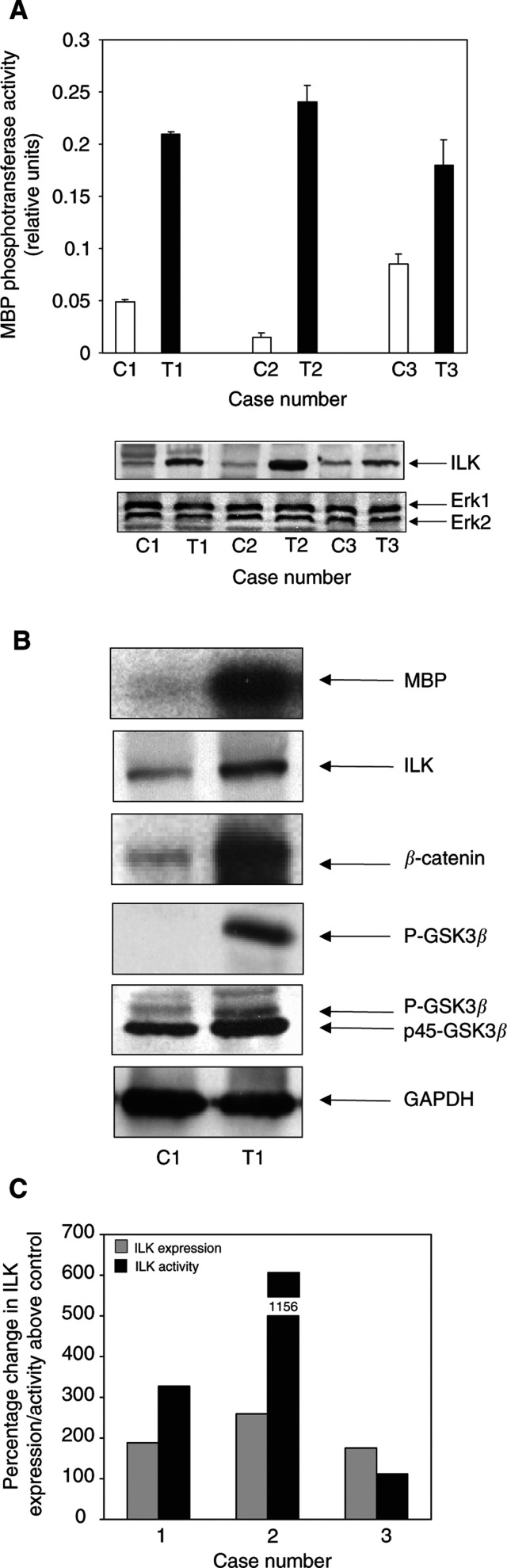
ILK signalling is dysregulated in human colon cancer. (**A**) ILK activity is enhanced in colorectal cancers. Upper panel, ILK MBP phosphotransferase densitometry. Immunoprecipitated ILK MBP phosphotransferase performed in triplicate. Middle panel, anti-ILK immunoblot, examining the protein expression of ILK in the tumour and the corresponding control sample. Lower panel; anti-Erk1-CT immunoblot, examining the expression of Erk1 and Erk2 in the control samples *vs* the corresponding tumour. (**B**) Effects of ILK on downstream targets. ILK activity is increased in the polyp compared with its respective control (representative autoradiogram). Anti-ILK immunoblot, examining expression of ILK. Anti-*β*-catenin immunoblot, examining the expression of *β*-catenin. Anti-P-GSK3*β* immunoblot, examining phosphorylation status of GSK3*β*. Anti-GSK3*β* immunoblot, examining expression of GSK3*β*. Anti-GAPDH immunblot, used as an internal control for experiments. The results are representative for the 24 cases, which displayed increases in ILK activity. (**C**) Correlation between the expression and activity of ILK in colonic tumours. The band intensities were quantitated as outlined in the Materials and Methods section. The values are represented as a percentage change in activity/expression above the corresponding control sample.

In our previous study, we demonstrated that overexpression of ILK in colonic polyps coincided with an increase in the immunoprecipitated ILK MBP phosphotransferase activity (Marotta *et al*, 2001). To determine whether ILK activity was similarly affected in human colon cancer, we characterised the biochemical activity in a total of 38 cases (16 cases in which metastatic deposits were present in the regional lymph nodes; an additional 22 cases with no lymphatic invasion). The representative data (Figure 4A

) indicate that changes in the expression of ILK (lower panel) coincided with changes in the MBP phosphotransferase activity in the cancerous lesions when compared to the normal adjacent control tissue. Two- to nine-fold increases in ILK activity were evident in 24 out of the 38 cases (63%). To determine whether there was a direct correlation between ILK expression and activity levels, the percentage change in the ILK activity above the control was compared with the percentage change in the ILK expression above the control (see Figure 4C). Although both displayed increased levels, a direct correlation was not apparent between them. It is worth adding that there were no measurable differences in the ILK protein expression between primary tumours with or without positive lymph nodes. Importantly, changes in the expression and activity of ILK appeared to be independent of changes in the protein expression of Erk1 and Erk2 in the same lesions. These results are in keeping with a number of reports that have examined the relative expression and activity of Erk1 and Erk2 in human malignancies such as colonic and pancreatic cancer (Attar *et al*, 1996; Yip-Schneider *et al*, 1999).

Since ILK has been shown to regulate GSK3*β* activity (Delcommenne *et al*, 1998) as well as modulate the subcellular distribution of *β*-catenin (Novak *et al*, 1998), we wanted to determine whether changes in ILK activity correlated with effects on these downstream targets *in vivo*. For the representative patient (Figure 4B), changes in the MBP phosphotransferase activity appeared to coincide with not only the overexpression of the ILK protein but as well with the stabilisation of *β*-catenin. Changes in the expression of the latter are not surprising, since approximately 85% of all sporadic colorectal cancers are believed to arise because of mutations in the APC gene (Mei *et al*, 1999). Interestingly, we observed an impressive increase in the phosphorylation of GSK3*β* at Ser-9 by immunoblotting with a phosphospecific GSK3*β* Ser-9 antibody as well as by retardation in the electrophoretic mobility of the GSK3*β* protein itself. Phosphorylation of GSK3*β* at Ser-9 coincides with a decrease in its phosphotransferase activity. These findings consolidate our preliminary data in colonic polyposis (Marotta *et al*, 2001) and indicate that this dysregulation is a stable reproducible event in multistage colon carcinogenesis. Additionally, elevated expression and activity of ILK appeared to be associated with an increase in the protein expression of Lef-1 (data not shown), a downstream target of ILK (Novak *et al*, 1998). The expression of GAPDH was assessed to control for protein loading.

There are a number of reports, which indicate that Ser473 of PKB is phosphorylated by an array of protein kinases including Mapkapk-2, PDK1/PRK2 and PKC. ILK has also been described as the putative PDK2 that phosphorylates this site on PKB. In this regard, ILK has been demonstrated to phosphorylate Ser473 *in vitro* and *in vivo*. Furthermore, modulation of ILK activity with a specific inhibitor (KP-SD1) has been reported to coincide with a decrease in Ser473 phosphorylation as well as modulate the activity of the respective kinase. Transient and stable overexpression of the ILK protein is known to correlate with an increase in not only Ser473 phosphorylation but also the biochemical activity of PKB.

To determine if changes in ILK expression resulted in changes in the phosphorylation status of PKB, we examined Ser473 phosphorylation by immunohistochemical analysis in the 16 cases in which metastatic lesions were present. The results, which are presented in Table 1

**Table 1:** Relative PKB Ser473 phosphorylation staining intensity

**Case number**	**Control**	**Primary cancer**	**Metastatic lesion**
3	1	1	1
4	1	1	1
6	1	2	2
8	1	1	1
9	1	1	1
12	1	1	1
15	1	1	1
22	1	1	1
23	1	1	1
25	1	1	1
26	1	1	2
27	1	2	2
32	1	1	1
33	1	1	1
35	1	1	1
38	1	1	1

Tissue sections corresponding to either control, primary cancer or metastatic lesion were stained as outlined in the Materials and Methods section.

, indicate that changes in PKB Ser473 phosphorylation occur infrequently in this disease based upon the intensity of the chromagen. These results are further corroborated by evidence indicating that there were no significant differences in immunoprecipitated PKB HH2B phosphotransferase activity between the control and tumour samples analysed (data not shown). These findings in colon cancer are supported by our recent findings in human breast cancer, which demonstrated that there were no statistically significant differences in PKB activity between the control and tumour samples analysed (Salh *et al*, 2002). To add further support, mammary tumours induced by specific overexpression of ILK correlated with a dramatic increase in the Ser-9 phosphorylation of GSK3*β*. However, only modest differences in the Ser473 phosphorylation status of PKB were observed in this animal model (White *et al*, 2001). Thus, it appears that ILK is more likely to regulate GSK3*β* activity directly *in vivo*, and dysregulation of this nexus, rather than PKB, might have an important role in epithelial-derived tumour growth and survival. It is also possible, however, that only modest changes in PKB activity are required for the antiapoptotic effect this kinase provides.

## DISCUSSION

In the present study, we report for the first time that the expression of ILK is significantly increased in sporadic colon cancer and metastatic deposits in regional lymph nodes, thus substantiating our original findings in colonic polyposis. We also show that increased immunoprecipitated ILK MBP phosphotransferase activity was evident in 63% of the cases. In addition to this, we demonstrate that elevated ILK expression and increased MBP phosphotransferase activity coincide with effects on downstream targets of ILK signalling such as GSK3*β* phosphorylation. Based upon the data presented here and in keeping with the ‘just right’ model for colorectal carcinogenesis, which states that specific APC genotypes are selected during tumour formation on the basis of the specific level of residual *β*-catenin downregulating activity that is retained, additional signals are likely required for the development of human colon cancer (Albuquerque *et al*, 2002). Perhaps ILK, via its effects on Wnt signalling, acts in concert with the loss of APC function to facilitate disease progression. It is worth adding that a fine balance must exist between those signals that influence Wnt signalling in a positive *vs* a negative manner since excessive accumulation of *β*-catenin has been reported to coincide with the induction of apoptosis (Kim *et al*, 2000).

One of the important observations made in these studies was the identification that GSK3*β* is phosphorylated at Ser-9, which is indicative of its inhibition. This could be of primary importance as it suggests that (pre)malignant cells retain the ability to modulate signalling pathways which ultimately regulate the subcellular distribution of *β*-catenin and that this regulation is probably a consequence of the ‘second hit’ in the wild-type APC allele (Albuquerque *et al*, 2002). This is in agreement with the ‘just right’ model. It is possible that ILK-mediated inhibition of GSK3*β* could destabilise the formation of the ‘destruction complex’ since phosphorylation of axin and APC by GSK3*β* is said to favour the formation of the complex (reviewed in Fodde *et al*, 2001). In addition to this, inhibition of GSK3*β* by ILK could tip the scale in favour of enhanced growth. Overexpression of ILK has been shown to promote anchorage-independent cell cycle progression, which is likely mediated by the upregulation of Tcf4-dependent gene transcription as well as by the inhibition of GSK3*β*. Interestingly, GSK3*β* is known to phosphorylate cyclin D1; phosphorylation is essential for degradation of the latter by the ubiquitin–proteasomal complex (Diehl *et al*, 1998). It is well established that the levels of cyclin D1 are increased in this disease (Kristt *et al*, 2000; Utsunomiya *et al*, 2001). In addition to the putative effects of GSK3*β* on growth, inhibition of this kinase could favour cell survival. A number of studies have shown that GSK3*β* can modulate apoptosis (Frame and Cohen, 2001; King *et al*, 2001).

As outlined above, 63% of the cases in which the biochemical activity of ILK was assessed displayed changes in the MBP phosphotransferase activity, whereas all of the lesions evaluated using immunohistochemistry demonstrated significant changes in ILK expression. This discrepancy between the expression and activity of ILK could be attributable to various factors involved in tissue sampling, such as the extent of tumour vascularisation and time of harvesting (normally less than 2 h) or even quite possibly because of differences in the proposed etiological pathways, that is, MIN *vs* CIN. However, in conjunction with our previous findings in FAP, it is plausible to assume that overexpression of and increased activity of ILK could be an important event not only in the initiation of colorectal carcinogenesis but as well in the progression of the disease. Support for the role of ILK in carcinogenesis is provided by elegant recent work, which investigated mammary epithelial-specific expression of ILK. The results from these studies indicated that overexpression of ILK not only resulted in the formation of multiple hyperplastic foci but as well a number of the transgenic mice developed focal mammary tumours (White *et al*, 2001).

Since ILK has been reported to enhance tumour cell invasion (Troussard *et al*, 2000), we postulated that ILK expression might be further dysregulated during the metastatic process. Surprisingly, the data from these studies indicated that there were no significant differences in the expression of ILK between the primary tumours and the metastatic deposits located within the regional lymph nodes. These data, in conjunction with our previous findings in colorectal adenomas from patients with FAP, suggest that changes in the expression of ILK occur very early during the development of colon cancer. It is plausible to assume, however, that although there were no significant differences in the expression of ILK between the primary and metastatic deposits, perhaps the activity of ILK is dysregulated at the invasive front. In support of this and in conjunction with the known effects of ILK on Tcf-dependent gene transcription, dramatic shifts in the subcellular distribution of *β*-catenin were shown to occur at the leading edge of the tumour (Kirchner and Brabletz, 2000; Jung *et al*, 2001). Furthermore, the tumour invasive front exhibits an epithelial–mesenchymal transition, as well as a loss of E-cadherin expression (Brabletz *et al*, 2001). Notably in this regard, over-expression of ILK has been reported to result in the downregulation of E-cadherin (Novak and Dedhar, 1999), whereas administration of a selective inhibitor of ILK has been reported to result in the induction of E-cadherin expression (Tan *et al*, 2001). Certainly, ILK has been reported to promote epithelial to mesenchymal transition in mammary epithelium (Somasiri *et al*, 2001). In order to specifically address the role of ILK activity at the invasive front, the development of specific antibodies capable of detecting the activated form of ILK would be required. We were unable to demonstrate any significant changes using an antibody specific for the activated form of PKB.

In summary, we suggest that dysregulation of ILK signalling is an important early event in the genesis of human colon cancer. Furthermore, with the recent surge in the development of specific inhibitors to protein kinases, we propose that an inhibitor to ILK should be explored as a possible novel strategy for either the treatment and/or prevention of this disease. An inhibitor to this protein kinase could prove to be as efficacious as the tyrosine kinase inhibitor STI-571, which is used in the treatment of chronic myeloid leukaemia (Thiesing *et al*, 2000; Joensuu *et al*, 2001). Moreover, administration of an ILK inhibitor in combination with other chemotherapeutic drugs might prove to be as useful as the EKI-569 (a reversible inhibitor of the EGF receptor tyrosine kinase) sulindac combination (Torrance *et al*, 2000). Currently, we are evaluating whether changes in ILK expression/activity occur predominantly in CIN *vs* MIN lesions. As well, we are attempting to unravel the mechanism by which ILK is dysregulated in colon cancer. Undoubtedly, this information could provide further insight into the underlying mechanisms involved in the initiation of carcinogenesis within the colon, as well as enhance our understanding of the ‘just right’ model for carcinogenesis.
